# Prehospital Fibrinolysis Therapy in Acute Myocardial Infarction: A Narrative Review

**DOI:** 10.7759/cureus.52045

**Published:** 2024-01-10

**Authors:** Mahmoud S Alsomali, Mazen A Alateeq, Saleh E Abuzaid, Abdulrahman M AlTurki, Yaser A Alnahdi, Mohammed A Koshan, Abdulrahman Musaad A Alsamih, Abdulaziz M Alsaif, Shahd Alharbi, Maha Abdullah S Alharbi, Rawshan Khalid A Alyoubi, Alhassan Ali A Alghamdi, Eman Fahad M Alanazi, Reem Saud H Alqahtani

**Affiliations:** 1 Emergency Medicine, King Fahad Medical City, Riyadh, SAU; 2 Emergency Medicine, Prince Sultan Military Medical City, Riyadh, SAU; 3 Emergency Medicine, King Abdulaziz University Hospital, Jeddah, SAU; 4 Clinical Sciences, College of Medicine, Dar Al Uloom University, Riyadh, SAU; 5 General Practice, King Faisal University, Alahsa, SAU; 6 General Practice, Al-Rayan Colleges, Al-Madinah Al-Munawwara, SAU; 7 Medicine, Al-Rayan Colleges, Al Madinah, SAU; 8 General Practice, National Guard Hospital, Jeddah, SAU; 9 General Practice, Primary Health Care 2 (PHC 2), Albaha, SAU; 10 General Practice, King Saud Bin Abdulaziz University for Health Sciences, Riyadh, SAU; 11 General Practice, King Khalid University, Abha, SAU

**Keywords:** tenecteplase (tnk), inj reteplase, alteplase (tpa), streptokinase, prehospital fibrinolysis, myocardial infarction, fibrinolytic therapy

## Abstract

Acute myocardial infarction is a fatal condition. Acute myocardial infarction requires appropriate timely reperfusion therapy to improve the outcomes. Fibrinolysis and percutaneous coronary intervention are the cornerstone strategies for managing such cases. In this review, our objective is to summarize the available evidence concerning the administration of prehospital fibrinolysis and its impact on patient outcomes in patients with acute myocardial infarction. We conducted a comprehensive literature search across PubMed, Cochrane Library, Scopus, and Web of Science databases. Our search strategy included the following terms: “Prehospital,” “EMS,” “Emergency Medical Services,” “ambulance,” “Fibrinolytic Therapy,” “alteplase,” “streptokinase,” “reteplase,” “tenecteplase,” “Acute Myocardial Infarction,” and “patient outcomes.” We found prehospital administration of fibrinolysis may improve the outcomes and decrease the mortality rate. We found that some recommendations were to use prehospital fibrinolysis only if the percutaneous coronary intervention was not accessible within two hours. Additionally, we discussed recommendations to use newer prehospital fibrinolysis as they have better efficacy and safety outcomes. In conclusion, prehospital fibrinolysis decreases the total ischemic time and improves outcomes in acute myocardial infarction patients when timely percutaneous coronary intervention is not available. The guidelines strongly recommend it when the anticipated time for percutaneous coronary intervention exceeds two hours. Ongoing research optimizes patient selection, treatment tools, and prehospital systems of care.

## Introduction and background

Acute myocardial infarction (AMI) ranks as a top cause of death in developed countries, affecting 3.8% of individuals less than 60 years and 9.5% of those aged 60 years and above [1]. Therefore, AMI requires prompt reperfusion therapy to improve clinical outcomes [2]. According to clinical guidelines, percutaneous coronary intervention (PCI) is the optimal first-line intervention for reperfusion in AMI [3-5]. When rapid access to PCI is unavailable, fibrinolysis therapy remains the preferred method for establishing timely reperfusion, according to the guidelines recommended for many patients [6]. A highly clinical and significant improvement is obtained after the implementation of fibrinolysis drugs within two to six hours [7,8]. Consistent with previous studies, fibrinolysis is administered after hospital arrival in the emergency department. However, prolonged time to establish a diagnosis and treatment initiation correlate with worse prognoses [9]. Welsh et al. illustrated the advantage of prehospital fibrinolysis in shortening the total ischemic time by administering fibrinolysis agents in the ambulance before hospital arrival. It augments reperfusion rates while shortening total ischemic time [10]. In addition, preclinical studies have demonstrated the capacity of fibrinolysis to restore coronary flow when administered before arrival at definitive care [11].

Highlighting the crucial importance of early intervention, particularly in prehospital scenarios, Table 1 offers treatment methods for AMI [5,6,8,10]. It compares prehospital fibrinolysis therapy, in-hospital fibrinolysis therapy, and PCI, providing key details in the form of treatment initiation times and potential side effects. This information helps to illuminate the effectiveness of these interventions. Fibrinolysis therapies within two hours of symptom onset reduced five-year mortality versus later interventions. As in prior studies, decreased clinical effectiveness was correlated with later treatment times. While reperfusion strategies yield comparable outcomes, the benefit of reperfusion diminishes sharply with delays exceeding 60 mins from the first medical contact to coronary reopening [12]. This review will provide a comprehensive summary of the current literature to evaluate how far prehospital fibrinolysis has come, clarify remaining uncertainties, and outline future research priorities.

**Table 1:** Acute myocardial infarction treatments: time to initiation, effectiveness, and side effects.

Treatment Type	Time to Initiation	Effectiveness	Side Effects
Prehospital Fibrinolysis Therapy	Within 1 hour of the first medical contact	Can restore blood flow in around 50-60% of patients	Common side effects include bleeding, especially at the site of injection. Less common but serious side effects include stroke and other bleeding complications
In-Hospital Fibrinolysis Therapy	Within 30 mins of hospital arrival	Similar to prehospital therapy but the delay may reduce the effectiveness	Similar to prehospital therapy including bleeding and stroke
Percutaneous Coronary Intervention (PCI)	<60 mins (since diagnosis) in PCI-capable centers and <90 mins in non-cabable ones.	Is generally more effective than fibrinolysis therapy9, with success rates often over 90%	Complications can include bleeding, especially at the site of catheter insertion, damage to the blood vessels, arrhythmias, contrast-induced acute kidney injury, and, rarely, stroke

## Review

Fibrinolysis therapy

Fibrinolysis therapy is a medical treatment designed to dissolve blood clots that have obstructed the flow of blood in the circulatory system [13]. This process helps to restore normal blood flow and minimize tissue damage [13]. Fibrinolysis therapy is commonly used in the management of conditions such as AMI, stroke, and pulmonary embolism, where rapid restoration of blood flow is critically important (Table 2) [14,15]. The therapy, however, has to be used wisely and according to guidelines, as it comes with the risk of causing significant bleeding [13].

**Table 2:** Different fibrinolysis agents used in prehospital settings.

Fibrinolysis Agent	Dosage	Effectiveness	Side Effects	Contraindications
Streptokinase	1.5 million units over 60 mins	Moderate	Bleeding, allergic reactions, fever	Previous exposure to streptokinase, recent surgery, bleeding disorders
Alteplase (tPA)	15 mg IV bolus, then 0.75 mg/kg (up to 50 mg) over 30 mins, then 0.50 mg/kg (up to 35 mg) over 60 mins	High	Bleeding, especially intracranial hemorrhage	Recent surgery, bleeding disorders, history of stroke
Reteplase (rPA)	Two IV boluses of 10 units each over 2 mins, 30 mins apart	High	Bleeding, especially intracranial hemorrhage	Recent surgery, bleeding disorders, history of stroke
Tenecteplase (TNK-tPA)	Single IV bolus dose, weight-adjusted (30-50 mg)	High	Bleeding, especially intracranial hemorrhage	Recent surgery, bleeding disorders, history of stroke

Rationale and importance of time

The importance of fibrinolysis therapy initiation in prehospital for patients experiencing AMI when timely PCI is not feasible is powered by robust evidence [3,16-22]. Several considerations support this approach to reperfusion therapy. Primarily, the duration between the onset of occlusion and reperfusion is critical in AMI, as the myocardium starts to undergo irreversible ischemic damage within minutes of occlusion [3,18]. Conventional in-hospital fibrinolysis often entails significant delays in treatment initiation as opposed to its counterpart, field administration by emergency medical services. Jordan et al. have indicated that prehospital fibrinolysis can curtail the average call-to-needle times by an interval ranging from 30 to 60 mins [16]. In the CAPTIM trial, Bonnefoy et al. demonstrated that early prehospital fibrinolysis reduces 30-day mortality in myocardial infarction patients. Additionally, patients who received fibrinolysis within two hours of symptom onset showed an improvement in five-year survival rates [19]. Saeedi et al. found that thrombolytic therapy administered immediately after symptom onset and before hospital transfer reduced mortality by 36% (OR = 0.64, 95% CI: 0.45-0.91) [20]. In addition, the American Heart Association ensures on necessity for door-to-needle times to be less than 30 mins for in-hospital fibrinolysis in cases of AMI [21]. In the same context, every half-hour of delay in reperfusion therapy in the case of STEMI is associated with a roughly 10% higher mortality [22]. Additionally, studies have found that administering fibrinolysis therapy within the first hour can lead to a complete reversal of symptoms in about one-fourth of patients, evidenced by normalized ECG findings and no signs of heart muscle damage [22].

Implementation, challenges, and limitations of prehospital fibrinolysis therapy

Administration of prehospital fibrinolysis for patients suffering from ST-elevation myocardial infarction (STEMI) hinges on the diagnostic capabilities of emergency medical services during out-of-hospital care [23,24]. Several factors can interfere with the correct assessment and stratification of AMI risk outside a hospital environment. A key factor in managing patients with acute chest pain is the recording and correct interpretation of electrocardiograms (EKGs) by paramedics [25]. EKGs are a vital diagnostic tool for STEMI. However, the conditions under which they are performed during ambulance transport can increase their susceptibility to errors caused by movement or technical issues [24]. This potential for error can create ambiguity in the identification of STEMI, which can in turn affect the decision-making process for patient management [26]. In addition to these challenges, the unavailability of needed diagnosis modalities rather than ECG during emergency transport, which are not available in the ambulance [23]. Choosing patients for prehospital fibrinolysis and administering fibrinolysis according to guidelines is another complicated task faced the healthcare professionals. This process is challenging because of the limitations of out-of-hospital assessments [27].

Evidence supports prehospital fibrinolysis in literature

The efficacy of prehospital fibrinolysis for AMI and the impact of early treatment initiation have been the focus of extensive research. The rationale for delivering fibrinolysis therapy immediately upon hospital arrival is clear and simple: the faster blood flow is restored during an AMI, the better the patient's chances are for a favorable recovery, and the lower the risk of complications such as heart muscle damage.

This is supported by the systematic review conducted by McCaul et al. It highlights the advantages of prehospital fibrinolysis. Their findings suggest that not only does prehospital fibrinolysis quicken the start of treatment, but it also correlates with reduced mortality. This benefit becomes even more significant when treatment begins within the first two hours after the onset of symptoms [28].

Furthermore, another systematic review done by Sim et al. contributes to this evidence by demonstrating that patients receiving a pharmaco-invasive approach, initial fibrinolysis followed by PCI, experienced rapid reperfusion and better blood flow [29].

In 2020, Fazel and his colleagues contributed to these findings by showing that the pharmaco-invasive strategy led to fewer adverse clinical events compared to PCI. They concluded that where primary PCI is not immediately available, the pharmaco-invasive treatment is more effective and safer than both facilitated PCI and sole fibrinolysis therapy [30]. This ensures the important and pivotal role of prehospital fibrinolysis administration even if PCI is available to avoid adverse events that follow AMI.

Additionally, Stiermaier et al. support the initiation of fibrinolysis in a prehospital setting to achieve timely reperfusion [31]. Early fibrinolysis administration decreases the time to start treatment and enhances overall outcomes [31]. Given the long PCI-related delay, the significance of prehospital fibrinolysis cannot be ignored.

Doan et al. suggest that if medical or paramedical are capable of interpreting ECG, fibrinolysis therapy should be started within 10 mins of diagnosing STEMI in the prehospital [32]. This protocol ensures that reperfusion therapy commences promptly, optimizing the chances for a successful clinical outcome.

Effect of fibrinolysis on clinical outcomes

In AMI management, fibrinolysis therapy is a pivotal reperfusion modality when timely PCI is not feasible. The COVID-19 era infection control protocols led to notable delays in PCI, resulting in a shift towards fibrinolysis for STEMI management to enhance clinical outcomes, which were assessed in terms of efficacy and safety [33]. Outcomes for AMI patients who received fibrinolysis therapy were comparable across facilities with varying capabilities for PCI [34]. According to the CAMI registry, STEMI patients receiving fibrinolysis therapy not only achieved successful clinical reperfusion but also demonstrated a more favorable two-year survival rate than those with unsuccessful fibrinolysis [35].

Kocayigit et al. highlighted that, in the elderly STEMI patients, fibrinolysis therapy’s short-term effects paralleled those of PCI, considering adverse events like reinfarction, major bleeding events, in-hospital mortality, and six-month mortality rates, although they observed an increase in one-year mortality within the fibrinolysis group [36]. Furthermore, a meta-analysis encompassing six randomized trials revealed that initiating fibrinolysis prior to hospital arrival cut down the odds of mortality by 17% compared to fibrinolysis administration in-hospital [37].

Prehospital fibrinolysis is validated as a safe and effective approach, significantly reducing short-term mortality in AMI patients [38]. Additional study has affirmed that the in-hospital mortality rates for STEMI patients receiving fibrinolysis are similar to those undergoing primary PCI [39].

Guidelines recommendations

The American Heart Association (AHA) and the American College of Cardiology (ACC) have advocated for the use of fibrinolysis before a patient arrives at the hospital for those suffering from myocardial infarction with ST-segment elevation (STEMI) when timely PCI is not available [21]. The two organizations have given a Class I recommendation for this strategy, stressing that it significantly shortens the time from when symptoms start to when treatment is given, compared to administering the treatment after the patient has been admitted to the hospital [21,23]. The guidelines suggest that a reduction of 30 mins in the time it takes to administer fibrinolysis treatment can lead to significant improvements in patient survival [23]. This support aligns with newer fibrinolysis drugs like tenecteplase, which are preferred due to their safety, effectiveness, and ease of administration [15,23]. They also highlight the necessity of strictly following treatment protocols to optimize the balance between the benefits of quicker restoration of blood flow and the risks of bleeding complications [23]. The European Society of Cardiology (ESC) also supports a Class I recommendation for administering fibrinolysis before hospital arrival when it is not feasible to perform PCI as soon as possible since diagnosis [40]. The ESC also suggests using newer fibrinolysis drugs like tenecteplase and emphasizes the necessity of strict protocols for choosing patients, delivering the drug, and transporting patients to hospitals capable of performing PCI to ensure the benefits are maximized while the risks are minimized [3,40].

Although the World Health Organization (WHO) does not have specific clinical practice guidelines that address the use of fibrinolysis before hospital arrival for STEMI, it does recommend early restoration of blood flow within 12 hours of symptom onset as a crucial part of treating acute coronary syndrome (ACS) [41]. The WHO supports the use of ECGs before hospital arrival and telemedicine/telecardiology to assist with early diagnosis by healthcare providers on the scene [41,42].

Future direction

Future research should focus on developments of decision support tools powered by artificial intelligence, which could speed up diagnosis and the choice of treatment. In addition, enhanced telemedicine communication between the first healthcare providers and teams based in the hospital will pave the way for rapid restoration of blood flow and improvement in patient outcomes [42].

## Conclusions

Prehospital fibrinolysis plays an essential role in the management of patients with STEMI. In such emergencies, where every second counts, the administration of fibrinolysis therapy before hospital arrival can significantly improve patient outcomes. The key benefits of this early fibrinolysis include reduced time to reperfusion, lowered mortality, and improved survival rates, particularly when timely PCI is not feasible. Current guidelines recommend prehospital fibrinolysis as a preferred treatment when PCI is delayed more than two hours.
